# ^MicroRNA 200c-3p regulates autophagy via upregulation of endoplasmic reticulum stress in PC-3 cells^

**DOI:** 10.1186/s12935-017-0500-0

**Published:** 2018-01-03

**Authors:** Eun Jung Sohn

**Affiliations:** Peripheral Neuropathy Research Center, Department of Physiology, College of Medicine, Dong-A University, Dongdaesin-Dong, Seo-Gu, Busan, 602-714 South Korea

**Keywords:** miR-200c-3p, ER stress, Autophagy, LC3

## Abstract

**Background:**

Autophagy is a response to cellular and environmental conditions and facilitates cell survival. Here, we investigated the role of ectopic expression of microRNA (miRNA) 200c-3p in autophagy.

**Methods:**

miRNA mimics were used to overexpress miRNAs. Quantitative real-time polymerase chain reaction (RT-qPCR) was performed to analyze miRNA expression. RT-qPCR and western blotting were performed to determine the expression levels of inositol requiring protein-1 (IRE1α), activating transcription factor-6 (ATF6), C/EBP homologous protein (CHOP), and light chain-3 (LC3).

**Results:**

Western blotting and RT-qPCR analysis revealed that ectopic expression of miR-200c-3p increased the expression of IRE1α, ATF6, and CHOP in PC-3 prostate cancer cells. Furthermore, the level of miR-200c-3p was enhanced by treatment with the endoplasmic reticulum (ER) stress inducer thapsigargin. In addition, ectopic expression of miR-200c-3p led to an increase in LC3-II expression, and formed puncta of green fluorescent protein-fused LC3-II in PC-3 cells. Interestingly, starvation stress induced by Hank’s balanced salt solution buffer increased the level of miR-200c-3p and conversely miR-200c-3p inhibitor blocked the increased expression of LC3-II induced by starvation in PC-3 cells. In addition, silencing of IRE1α by transfection of short interfering RNA attenuated the expression of LC3-II induced by upregulation of miR-200c-3p in PC-3 cells.

**Conclusions:**

Overall, our findings suggest that miR-200c-3p regulates autophagy via upregulation of ER stress signaling.

## Background

Autophagy is characterized by the degradation of cellular components in lysosomes [1]. The initial step of autophagy involves the formation of dysfunctional organelles, misfolded/aggregated proteins, or autophagosomes [2, 3]. In the late stage, autophagosomes fuse with lysosomes to generate autolysosomes and substrates are degraded by lysosomal hydrolases. This programmed cell destruction plays a role in cancer, cell death, survival, and adaptive responses [4–7].

Endoplasmic reticulum (ER) stress, which functions in protein folding, is considered an inducer of autophagy [8]. Intracellular and extracellular stimuli can substantially affect ER functions, leading to the accumulation of unfolded or misfolded proteins in the ER lumen [9]. To avoid cell damage, accumulation of unfolded or misfolded proteins activates the unfolded protein response (UPR), which involves the three major transducers of ER stress, activating transcription factor-6 (ATF6), inositol requiring protein-1 (IRE1α), and protein kinase RNA-like ER kinase (PERK). ER stress can induce apoptosis by activating the UPR in cancer, and may be a target for anticancer treatment [10].

MicroRNAs (miRNAs) are small non-coding RNAs that serve as negative regulators of gene expression involved in cell growth, cancer, apoptosis, and aging [11, 12]. The role of miRNAs as novel regulators of autophagy and ER stress has been well documented [13, 14]. Thus, in this study, we investigated the role of miR-200c-3p in autophagy. Here, we demonstrate that overexpression of miR-200c-3p promotes ER stress signaling to induce autophagy via light chain-3 (LC3)-II activation and autophagosome formation in PC-3 prostate cancer cells.

## Materials and methods

### Cell culture

PC-3 cells were purchased from the American Type Culture Collection (Manassas, VA, USA) and cultured in RPMI 1640 medium supplemented with 10% fetal bovine serum (Welgene, Daegu, Korea), 2 μM l-glutamine, and penicillin/streptomycin (Invitrogen, Carlsbad, CA, USA) in a 5% CO_2_ atmosphere at 37 °C.

### Cytotoxicity assay

To assess cytotoxicity, PC-3 cells were transfected with miR-200c-3p mimics (Genolution, Korea). Two days after transfection, the cells were treated with or without 0.5 mM thapsigargin (TG) (Sigma, St. Louis, MO, USA) for 24 h and the 3-(4,5-dimethylthiazol-2-yl)-2,5-diphenyltetrazolium bromide (MTT) assay (Sigma) was performed according to the manufacturer’s instructions.

### Quantitative reverse transcription polymerase chain reaction (RT-qPCR) analysis

Total RNA from PC-3 cells transfected with control, miR-200c-3p mimic, or miR-200c-3p inhibitor was isolated using QIAzol (Invitrogen). One microgram of total RNA was used to generate complementary DNA (cDNA) by superscript reverse transcriptase (Invitrogen). RT-qPCR was performed with the LightCycler instrument (Roche Applied Sciences, Indianapolis, IN, USA) with the following primers: LC3-II-forward, 5′-GCC TTC TTC CTG CTG GTG AAC-3′ and reverse, 5′-AGC CGT CCT CGT CTT TCT CC-3′; Beclin 1-forward, 5′-GGA TGG ATG TGG AGA AAG GCA AG-3′ and reverse, 5′-TGA GGA CAC CCA AGC AAG ACC-3′; ATF6-forward, 5′-AACAAGACCACAAGACCAA-3′ and reverse, 5′-AGGAGGAACTGACGAACT-3′; C/EBP homologous protein (CHOP)-forward, 5′-CTCCTTCGGGACACTGTCCA-3′ and reverse, 5′-CTTTCTCCTTCATGCGCTGC-3′; PERK-forward, 5′-CGATGAGACAGAGTTGCGAC-3′ and reverse, 5′-TGCTTTCACGGTCTTGGTC-3′; eukaryotic initiation factor (eIF) 2α-forward, 5′-CTCTTGACAGTCCGAGGATC-3′ and reverse, 5′-GTATCCCAGCTGTGCCATCT-3′; and glyceraldehyde 3-phosphate dehydrogenase (GADPH)-forward, 5′-AGGGCTGCTTTTAACTCTGGT-3′; and reverse, 5′-CCCCACTTGATTTTGGAGGGA-3′.

### Western blotting

PC-3 cells were transfected with miR-135a, miR-1290, miR-200c-3p, miR-374b, miR-3195, and control mimic plasmids (Genolution). Two days after transfection, the cells were lysed in radioimmunoprecipitation buffer (50 mM Tris–HCl, pH 7.4, 150 mM NaCl, 1% NP-40, 0.25% sodium deoxycholic acid, 1 M EDTA, 1 mM Na_3_VO_4_, 1 mM NaF, and protease inhibitor cocktail). Protein samples were separated by sodium dodecyl sulfate polyacrylamide gel electrophoresis and electrotransferred onto a Hybond enhanced chemiluminescence (ECL) transfer membrane (Amersham Pharmacia, Piscataway, NJ, USA). After blocking, the membrane was incubated with the following primary antibodies: LC3-II (1:1000; #2775, Cell Signaling Technology, Danvers, MA, USA), PERK (1:1000; #5683, Cell Signaling Technology), IRE1α (1:1000; #3294, Cell Signaling Technology), 78 kDa glucose-regulated protein (GRP78) (1:500; #sc-376768, Santa Cruz Biotechnology, Santa Cruz, CA, USA), ATF6 (1:1000; #65880, Cell Signaling Technology), Beclin (1:1000, #3738, Cell Signaling Technology), and β-actin (1:5000; #4970, Cell Signaling Technology). After washing, the membrane was incubated with horseradish peroxidase-conjugated secondary anti-mouse or anti-rabbit antibodies (1:5000; AbD Serotec, Kidlington, UK). An ECL system (Amersham Pharmacia) was used to visualize the protein bands.

### Short interfering RNA transfection assay

PC-3 cells were transiently transfected with control, IRE1α, or PERK short interfering RNA (siRNA; Bioneer, Korea) using interferin transfection reagent (Polyplus-transfection Inc., New York, NY, USA). Briefly, the mixture of siRNA (40 nM) and interferin transfection reagent were incubated for 10 min, and added to PC-3 cells.

### miRNA transfection assay

miR-200c-3p and control mimic plasmids (200 nM; Genolution) were transfected into PC-3 cells using interferin transfection reagent (Polyplus-transfection Inc.) according to the manufacturer’s instructions. To evaluate the expression of miR-200c-3p, total RNA from PC-3 cells was isolated with QIAzol following treatment with TG (Invitrogen). To construct miRNA cDNA, a GenoExplorer miRNA cDNA kit (GenoSensor Corporation, Tempe, AZ, USA) was used according to the manufacturer’s instructions. To measure miRNA levels, RT-qPCR analysis was performed using the LightCycler instrument (Roche Applied Sciences). miRNA primers of miR-200c-3p were purchased from GenoExplorer (GenoSensor Corporation), and the U6 small nuclear ribonucleoprotein (snRNA) primer was used to normalize miRNA levels. Sequences of the U6 snRNA primers were as follows: forward, 5′-CGCTTCGGCAGCACATATAC-3′ and reverse, 5′-TTCACGAATTTGCGTGTCAT-3′.

### Immunofluorescence assay

PC-3 cells grown on LAB-TEK II chamber slides (Nalge Nunc International, Rochester, NY, USA) following transfection of miR-200c-3p mimic (100 nM) were washed and fixed with 4% paraformaldehyde in phosphate-buffered saline (PBS) for 20 min. Fixed cells were washed with PBS and permeabilized with 1% Triton-X 100 in PBS for 5 min. The primary antibody, anti-LC3-II (1:1000; Cell Signaling Technology), diluted in 1% bovine serum albumin in PBS, was added and incubated overnight at 4 °C. Fixed cells were washed and stained with the corresponding Alexa Fluor fluorescent antibody (1:2000; ab150077, Abcam, Cambridge, UK) for 30 min at room temperature. After washing, cell nuclei were counterstained with 1 μg/mL 4′,6-diamidino-2-phenylindole and mounted. Images were obtained using a Delta Vision imaging system (Applied Precision, Issaquah, WA, USA).

### Statistical analyses

Statistical analyses of the data were conducted using Prism software (La Jolla, CA, USA). All data were expressed as the mean ± standard error of the mean. Statistically significant differences between the control and treatments were determined by Student’s t test.

## Results

### Ectopic expression of miR-200c-3p induced ER stress markers

**Fig. 1:**
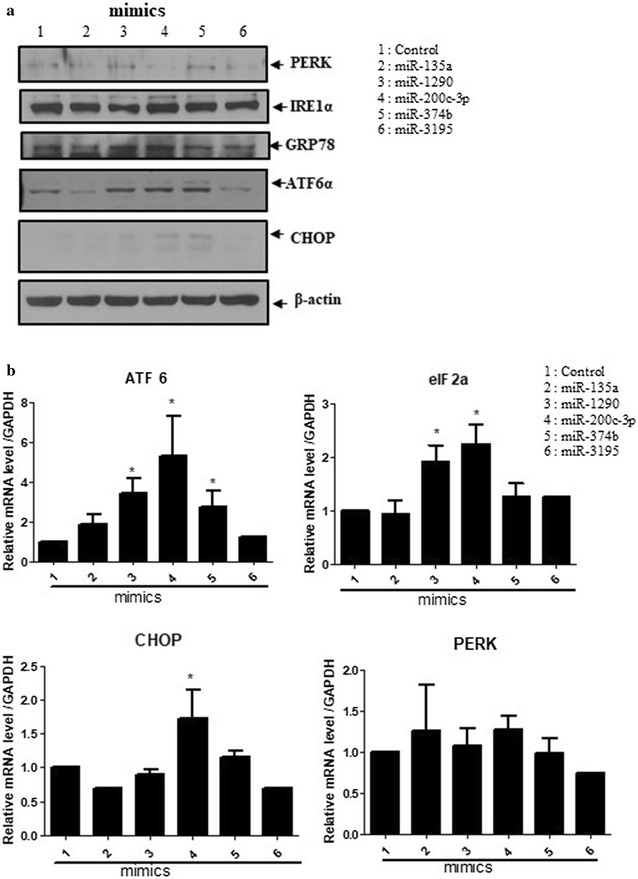
Ectopic expression of microRNA (miRNA)-200c-3p enhanced the levels of endoplasmic reticulum (ER) stress-related proteins. PC-3 cells were transfected with miR-135a, miR-1290, miR-200c-3p, miR-374b, miR-3195, or control mimics (200 nM). **a** Western blotting was used to determine the expression of protein kinase RNA-like ER kinase (PERK), inositol requiring protein-1 (IRE1α), 78 kDa glucose-regulated protein (GRP78), activating transcription factor-6 (ATF6), C/EBP homologous protein (CHOP), and β-actin. **b** Quantitative real-time polymerase chain reaction (RT-qPCR) was performed to determine the mRNA expression levels of ATF6, elF2α, CHOP, and PERK. Levels of glyceraldehyde 3-phosphate dehydrogenase (GAPDH) were used for normalization. 1, control; 2, miR-135a; 3, miR-1290; 4, miR-200c-3p; 5, miR-374b; and 6, miR-3195. Data are presented as the mean ± standard error of the mean (SEM) of triplicate samples. *p < 0.05

To determine whether miRNAs were involved in ER stress, we randomly selected several miRNAs, including miR-135a, miR-1290, miR-200c-3p, miR-374b, and miR-3195. After transfection with miRNA mimics, we determined the expression levels of the ER stress markers IRE1α, PERK, CHOP, ATF6, and GRP78. As shown in Fig. 1a, western blotting revealed that the levels of CHOP, ATF6, and IRE1α, but not GRP78 and PERK, were increased following transfection with miR-1290, miR-200c-3p, and miR-374b mimics. Consistent with this, RT-qPCR analysis showed that ectopic expression of the miR-200c-3p mimic in PC-3 cells increased the mRNA level of ATF6, elF2α, and CHOP, but not PERK (Fig. 1b), suggesting that miR-200c-3p positively regulates ER stress. Therefore, we focused on miR-200c-3p in this study.

### miR-200c-3p was regulated by ER stress

**Fig. 2:**
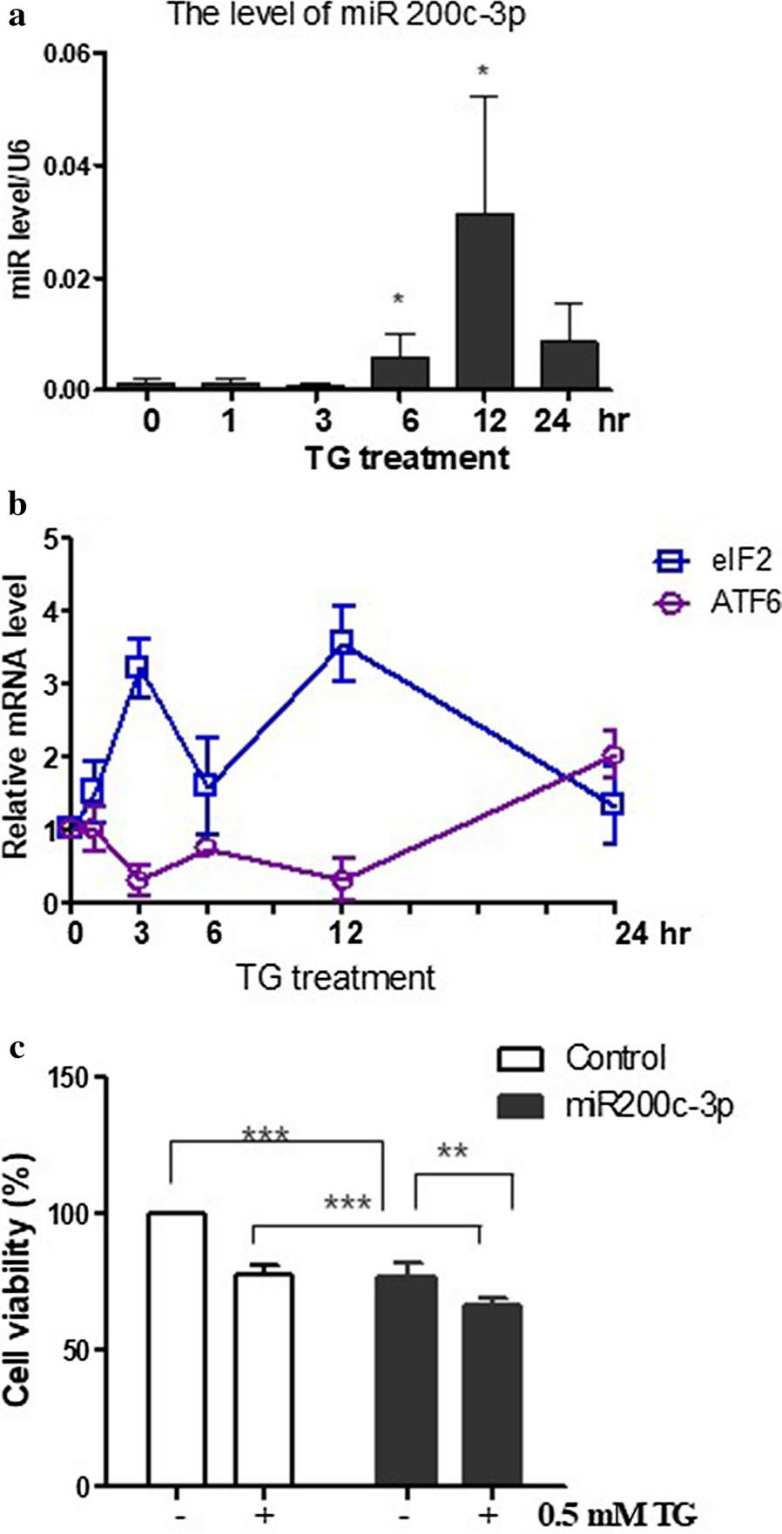
miR-200c-3p was associated with ER stress. **a** The level of miR-200c-3p was increased following thapsigargin (TG) treatment in PC-3 cells. After treatment with TG for 0, 1, 3, 6, 12, and 24 h, cells were lysed and complementary DNA was generated. RT-qPCR analysis was performed to determine the level of miR-200c-3p. U6 small nuclear ribonucleoprotein was used to normalize the expression of miR-200c-3p. **b** The expression levels of ATF6 and eukaryotic initiation factor (eIF)-2α were increased following TG treatment. RT-qPCR analysis was performed to evaluate the levels of ATF6 and elF2α. Levels of GAPDH were used for normalization. **c** Viability of PC-3 cells treated with TG in control or miR-200c mimics. Two days after transfection with miR-200c-3p mimic, 0.5 mM TG was added and cells were incubated for 48 h. The MTT assay was used to measure cell viability. Data are presented as the mean ± SEM of triplicate samples. ***p < 0.001, **p < 0.01, and *p < 0.05

To determine whether miR-200c-3p was regulated by ER stress, we treated PC-3 cells with the ER stress inducer TG. As shown in Fig. 2a, miR-200c-3p expression was determined by RT-qPCR in TG-treated PC-3 cells. After 6-h treatment with TG (0.5 mM), the level of miR-200c-3p was increased for 24 h (Fig. 2a). We also determined the expression of eIF2α and ATF6 mRNA as a positive control of ER stress (Fig. 2b). To determine the role of miR-200c-3p in cytotoxicity induced by ER stress, miR-200c-3p was transfected into cells, followed by treatment with TG for 48 h. As shown in Fig. 2c, the miR-200c-3p mimic enhanced the TG-induced cytotoxicity in PC-3 cells.

### Ectopic expression of miR-200c-3p induced autophagy in PC-3 cells

**Fig. 3:**
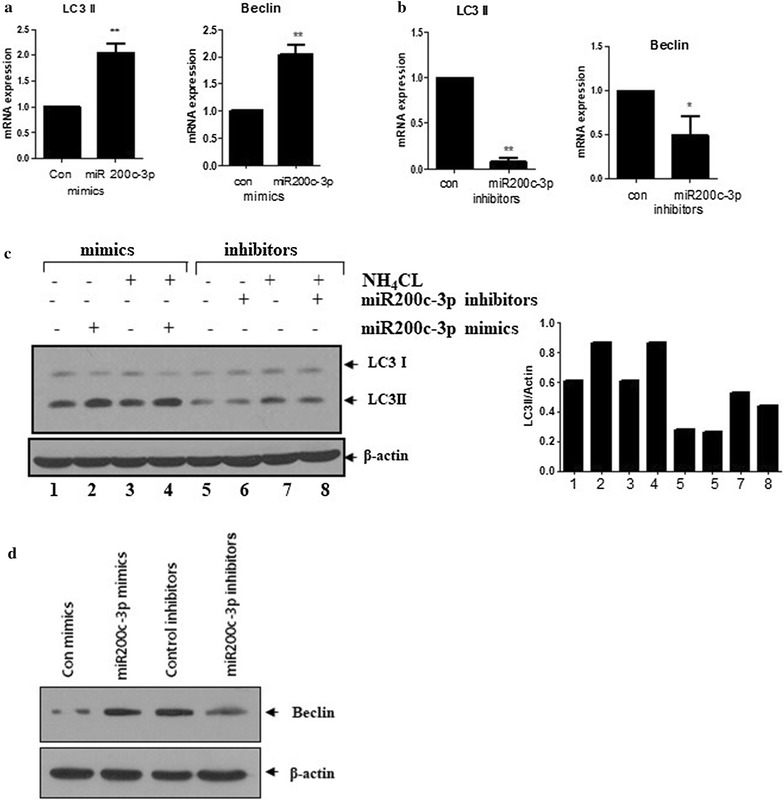
miR-200c-3p enhanced LC3-II expression in PC-3 cells. PC-3 cells were transfected with control, miR-200c-3p mimic (200 nM), or miR-200c-3p inhibitor (100 nM). At 48 h post-transfection of miR-200c-3p mimic **a** or miR-200c-3p inhibitor **b**, LC3-II and Beclin mRNA levels were determined by RT-qPCR. Levels of GAPDH were used for normalization. Data are presented as the mean ± SEM of triplicate samples. **p < 0.01 and *p < 0.05. **c** LC3-II expression was determined by western blotting in miR-200c-3p mimic- or inhibitor-transfected PC-3 cells in the presence or absence of NH_4_Cl. **d** Western blotting was performed following transfection with miR-200c-3p mimic or inhibitor to determine the levels of Beclin and β-actin

**Fig. 4:**
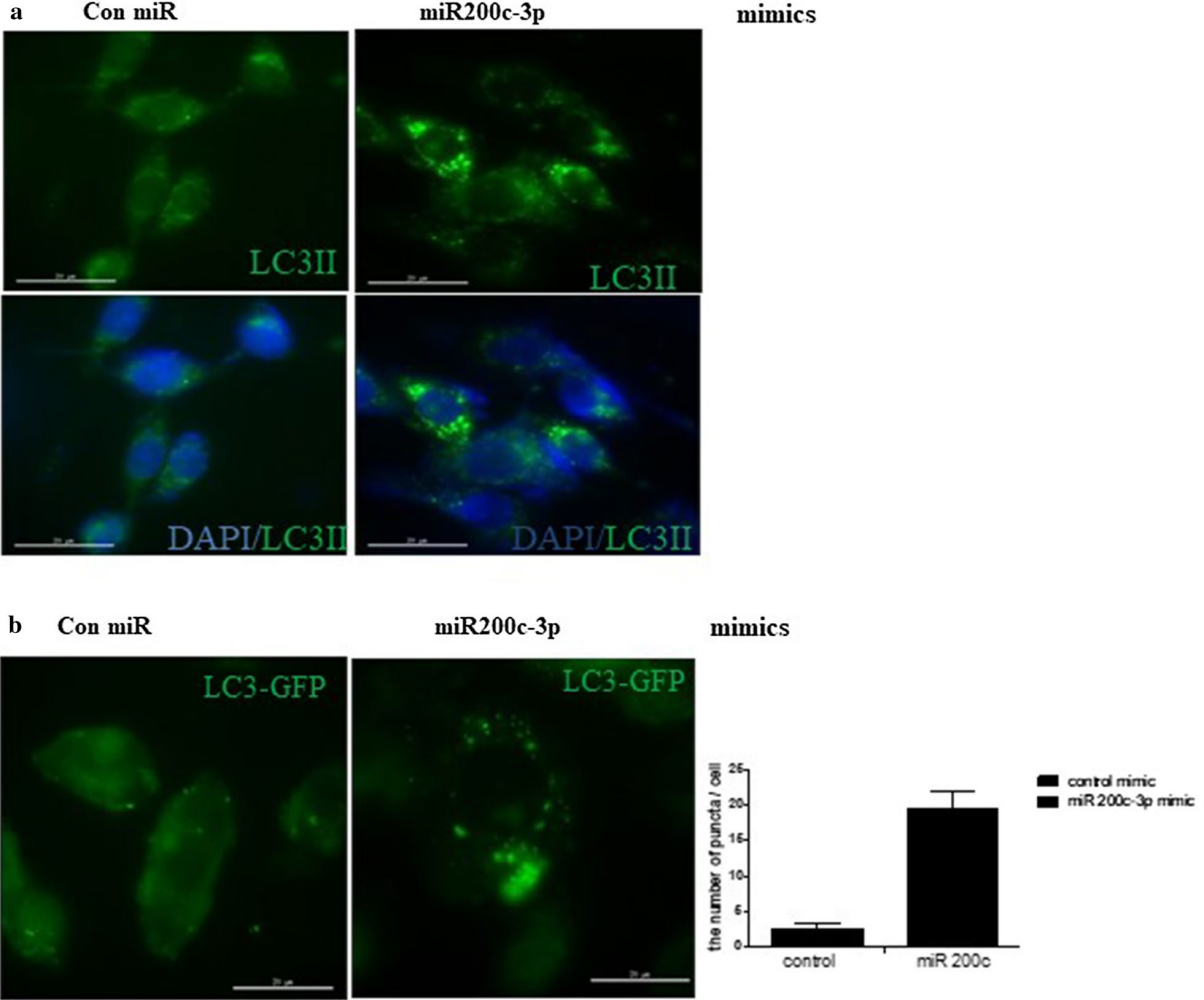
miR-200c-3p enhanced the formation of autophagosomes in PC-3 cells. **a** Endogenous expression of LC3-II in miR-200c-3p-transfected PC-3 cells. Following transfection of miR-200c-3p or control mimics, the cells were fixed and immunostained with LC3-II antibody. **b** Puncta formation of green fluorescent protein (GFP)-fused LC3-II plasmids in miR-200c-3p-transfected PC-3 cells. A GFP-fused LC3-II plasmid and miR-200c-3p mimic were cotransfected into PC-3 cells. At 48 h post-transfection, GFP-fused LC3-II expression was visualized by live cell microscopy. Data are presented as the mean ± SEM of triplicate samples. Scale bar = 20 μm

ER stress induces autophagy [8]. Therefore, we predicted that autophagy might be induced by ectopic expression of miR-200c-3p in PC-3 cells. RT-qPCR was performed following transfection of miR-200c-3p mimic or inhibitor in PC-3 cells to determine the effects on LC3-II and Beclin mRNA levels, which are markers of autophagy. As shown in Fig. 3, ectopic expression of miR-200c-3p increased the mRNA levels of LC3-II and Beclin, while the miR-200c-3p inhibitor attenuated their expression (Fig. 3a, b). Next, western blotting was performed to evaluate the conversion of LC3-I to LC3-II following overexpression of miR-200c-3p. Similar to the RT-qPCR results, western blotting revealed that ectopic expression of the miR-200c-3p mimic increased LC3-II expression, while the miR-200c-3p inhibitor slightly attenuated LC3-II expression (Fig. 3c). In addition, we treated miR-200c-3p mimic- or inhibitor-transfected PC-3 cells with or without NH_4_Cl, an inhibitor of acidification that blocks fusion of the lysosome. This treatment had no effect on LC3-II expression compared with NH_4_Cl alone, suggesting that miR-200c-3p might block autophagic flux (Fig. 3c). Furthermore, we observed that the miR-200c-3p mimic increased protein levels of Beclin while the miR-200c-3p inhibitor attenuated the level of Beclin (Fig. 3d). Next, we evaluated autophagic activity by fluorescence microscopy and found that overexpression of the miR-200c-3p mimic resulted in the formation of puncta of endogenous LC3-II outside the autophagosomes (Fig. 4a). Additionally, following transfection with a green fluorescent protein (GFP)-fused LC3-II plasmid, the formation of GFP-fused LC3-II puncta was increased in miR-200c-3p mimic-transfected PC-3 cells (Fig. 4b).

**Fig. 5:**
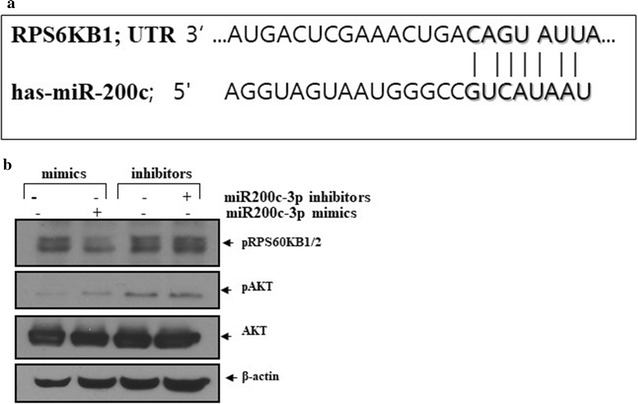
Ectopic miR-200c-3p suppressed ribosomal protein S6 kinase (RPS60KB). **a** Schematic representation of predicted functional interactions between miR-200c-3p and the seed sequences (bold) in the 3′ untranslated region of RPS60KB (http://www.targetscan.org). **b** PC-3 cells were transfected with the control, miR-200c-3p mimic (200 nM), or miR-200c-3p inhibitor (100 nM). Western blotting was performed to measure phosphorylated RPS60KB1/2, AKT, pAKT, or β-actin levels

To discover additional players in the autophagy pathway, we evaluated ribosomal protein S6 kinase (RPS6K) because it is a downstream target of mammalian target of rapamycin complex 1 (mTORC1) and has been reported to be a negative regulator of autophagy [15]. We found a functional interaction between miR-200c-3p and RPS6KB1 using the bioinformatics tool Targetscan (Fig. 5a). To validate this, the expression of RPS6KB1/2 was determined following transfection with a miR-200c-3p mimic and inhibitor. As shown in Fig. 5b, western blotting revealed that the miR-200c-3p mimic attenuated the phosphorylation of RPS60KB while the miR-200c-3p inhibitor increased phosphorylation.

### Effect of starvation on the levels of miR-200c-3p

**Fig. 6:**
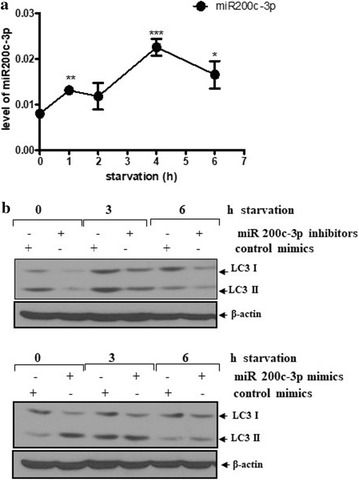
miR-200c-3p was increased by starvation and an miR-200c-3p inhibitor blocked LC3-II expression during starvation. **a** The levels of miR-200c-3p during starvation. PC-3 cells were incubated in Hank’s balanced salt solution (HBSS) for 0–6 h and the levels of miR-200c-3p were determined by RT-qPCR. **b** Blocking of LC3-II expression in miR-200c-3p-transfected PC-3 cells during starvation. Two days after transfection with the control, miR-200c-3p inhibitor, or miR-200c-3p mimic, cells were incubated in HBSS medium for 0, 3, and 6 h. Western blotting was performed to determine the levels of LC3-II and β-actin. Data are presented as the mean ± SEM of triplicate samples. ***p < 0.001, **p < 0.01, and *p < 0.05

To further investigate the role of miR-200c-3p in autophagy, nutrient starvation was induced in PC-3 cells for 2, 4, or 6 h using Hank’s balanced salt solution (HBSS) and the level of miR-200c-3p was examined by RT-qPCR, which showed that starvation led to an increase in the level of miR-200c-3p (Fig. 6a). To determine whether miR-200c-3p was responsive to autophagy induced by starvation, PC-3 cells were transfected with a control, miR-200c-3p mimic, or miR-200c-3p inhibitor, and 2 days after transfection, starvation by HBSS was induced for 3 or 6 h. After starvation for 3 h, the miR-200c-3p inhibitor blocked the increase in LC3-II compared with the control inhibitor, while the miR-200c-3p mimic increased LC3-II slightly (Fig. 6b).

### miR-200c-3p induced autophagy in an IRE1α-dependent manner

**Fig. 7:**
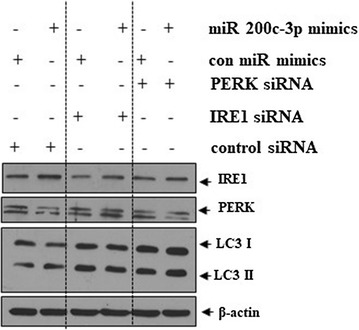
Silencing of IRE1α blocked the conversion of LC3-I to LC3-II in miR-200c-3p-transfected PC-3 cells. PC-3 cells were transfected with control, IRE1α, or PERK short interfering RNAs (siRNAs). The following day, control or miR-200c-3p mimics were transfected into siRNA-treated cells. One day after the second transfection, western blotting was performed to determine the levels of IRE1α, PERK, LC3-II, and β-actin

Several lines of evidence suggest that ER stress causes autophagy. Specifically, IRE1α and PERK are important mediators of ER stress-induced autophagy [16, 17]. To determine whether miR-200c-3p induced autophagy via ER stress, we transfected control or miR-200c-3p mimic in IRE1α- or PERK-silenced PC-3 cells with siRNA. As shown in Fig. 7, transfection of the miR-200c-3p mimic in control siRNA-treated cells enhanced the level of LC3-II, while LC3-II expression was attenuated in IRE1α-silenced PC-3 cells. However, there was no attenuation of LC3-II in PERK siRNA-treated PC-3 cells. Our results indicate that miR-200c-3p induces autophagy in an IRE1α-dependent manner.

## Discussion

The role of the miR-200 family in cancer has been extensively investigated. miR-200 family members are frequently downregulated in metastases, with low expression of miR-200 inducing aggressive, invasive, or chemoresistant phenotypes in several cancer types, including non-small-cell lung cancer and female reproductive cancers [18, 19]. miR-200 family members also regulate the epithelial-mesenchymal transition (EMT), which is important in the metastatic progression of many types of cancer. miR-200 family members have been shown to regulate the EMT via the transcriptional repressors ZEB1 and SIP1/ZEB2 [20–22]. Although the role of the miR-200c family in the EMT has been investigated extensively, its role in autophagy has not been studied. Here, we report a potential role for miR-200c-3p in autophagy-mediated ER stress in prostate cancer cells.

Evidence suggests that miRNA expression can regulate or be regulated by the UPR of the ER. In addition, ER stress induces miR-30c-2* [23], and reduces the expression of the miR-199a/214 cluster in human hepatocellular cancer [24]. Chitnis et al. reported that miR-211 was enhanced in mammary carcinoma in a PERK-dependent manner while increased expression of miR-211 attenuated the accumulation of CHOP [25]. A recent study reported that miR-200c exerted an anticancer effect via ER stress in H460 cells [26]. Similarly, in this study, we demonstrated that ectopic expression of miR-200c-3p resulted in increased expression of IRE1α, ATF6, and CHOP, and ER stress regulated the expression of miR-200c-3p. This suggests that miR-200-3p regulates or is regulated by ER stress.

Several studies have revealed that miRNAs are linked to autophagy, including evidence that miR-23b can sensitize pancreatic cancer cells to radiation treatment by blocking radiation-induced autophagy [27], and miR-155 and miR-31 inhibit interferon γ-induced autophagy [28]. Inhibition of miR-142-3p, miR-376A, and miR-376B induced autophagy-mediated starvation [29]. It is also known that miR-101 and miR-30a are potent inhibitors of autophagy [30, 31]. miRNAs are thought to be negative regulators of autophagy, with the exception of miRNA-155, which enhanced autophagy to eliminate intracellular mycobacteria [32] and miR-18a, which enhanced autophagy in colon cancer cells [33]. In this study, we demonstrated that overexpression of miR-200c-3p with a mimic increased the expression of LC3-II and the accumulation of LC3 puncta in PC-3 cells. In addition, the miR-200c-3p inhibitor blocked starvation-induced LC3-II expression. Thus, our data suggest that miR-200c-3p enhances autophagy in PC-3 cells.

## Conclusion

Overall, our data demonstrated that miR-200c-3p increased the expression of ER stress genes as well as the expression of LC3-II. Furthermore, the level of miR-200c-3p was increased in starvation-induced autophagy. Thus, our data suggest that miR-200c-3p-mediated autophagy, which is induced via ER stress, might be a potential target for the treatment of prostate cancer cells.
